# Exploring the Illness Experience of Patients with Central Nervous System Hemangioblastomas in Von Hippel–Lindau Disease: A Qualitative Study

**DOI:** 10.3390/healthcare14020275

**Published:** 2026-01-21

**Authors:** Mei-Fang Chuang, Pi-Hua Huang, Jing-Shan Huang, Chii Jeng

**Affiliations:** 1 School of Nursing, College of Nursing, Taipei Medical University, Taipei 110301, Taiwan; fang525100@gmail.com; 2 Department of Nursing, Cathay General Hospital, Taipei 106438, Taiwan; 3 Department of Nursing, St. Mary’s Junior College of Medicine, Nursing and Management, Yilan 266003, Taiwan; d432106008@tmu.edu.tw; 4 Department of Neurosurgery, Cathay General Hospital, Taipei 106438, Taiwan; jac3@cgh.org.tw

**Keywords:** Von Hippel–Lindau disease, hemangioblastoma, qualitative research

## Abstract

**Background/Objectives:** Von Hippel–Lindau (VHL) disease is a rare autosomal dominant hereditary disorder. Central nervous system hemangioblastomas are one of the most common tumor types associated with VHL disease. Although these tumors are histologically benign, delayed diagnosis and treatment may result in severe neurological dysfunction, permanent disability, and even death. However, little is known about the experiences of patients with VHL disease. The aim of this study was to gain a better understanding of the illness experiences and psychological responses of patients with VHL disease accompanied by central nervous system hemangioblastomas. **Methods:** A qualitative study based on a semi-structured guide was conducted. Twelve participants were recruited. Data were collected through face-to-face interviews and analyzed using the constant comparative method. **Results:** Four themes and their subthemes were identified: 1. powerlessness—unpredictable disease progression and uncontrollable continuity; 2. negative emotional experiences—guilt and self-blame, depression, and low self-esteem; 3. compromise—acceptance of fate, positive outlook, and sense of hope; and 4. persistent worry—worries about family members, anxiety regarding finances and employment, and uncertainty regarding the future. **Conclusions:** This study identified four major themes in the illness experiences of patients with VHL disease accompanied by central nervous system hemangioblastomas, which provided deep insights into the care needs of individuals with VHL disease. Healthcare providers should develop effective measures to enhance patients’ ability to maintain a good quality of life and confront the future with resilience.

## 1. Introduction

Von Hippel–Lindau (VHL) disease is a rare autosomal dominant hereditary disorder associated with mutations in the VHL gene located on the short arm of chromosome 3 (3p25.3). The VHL gene functions as a tumor-suppressor gene, and mutations inducing loss of function in this gene lead to impaired degradation of hypoxia-inducible factors, promoting abnormal cellular proliferation and tumor formation [1,2]. Studies conducted in European countries, such as Denmark, the UK, and Germany, reported incidences of VHL disease ranging from 1 in 27,300 to 1 in 42,897 individuals [1]. The global incidence of VHL disease is estimated to be approximately 1 in 36,000 individuals, with familial inheritance responsible for approximately 80% of cases and sporadic mutations responsible for the remaining 20% [3,4].

Central nervous system (CNS) hemangioblastomas are one of the most common tumor types associated with VHL disease, affecting 60–80% of patients. Although these tumors are histologically benign, delayed diagnosis and treatment may result in severe neurological dysfunction and permanent disability [5,6,7], rendering them a leading cause of mortality in patients with VHL disease. The diagnosis and surveillance of CNS hemangioblastomas in patients with VHL disease require a rigorous follow-up strategy; early detection of lesions can substantially reduce the risk of neurological complications and improve treatment outcomes. Nevertheless, delays in diagnosis and inadequate surveillance remain prevalent in clinical practice [8,9]. Although most patients present with symptoms of CNS hemangioblastomas at the age of 30 to 40 years, 29% of patients under the age of 18 years exhibit CNS hemangioblastomas as their initial manifestation, and 34% present with retinal lesions [10]. VHL disease imposes physiological and psychological burdens on patients and leads to substantial medical expenditures and caregiving demands, undermining patients’ financial stability and overall quality of life [11,12].

In Taiwan, the government enacted the Rare Disease and Orphan Drug Act in 2000, formally integrating rare diseases into the national health protection system. The Act seeks to promote the prevention, early diagnosis, and medical care of rare diseases, as well as to facilitate the development and accessibility of orphan drugs [13]. According to official records, only 43 cases of VHL disease were reported in Taiwan between July 2018 and December 2024, among whom seven patients subsequently died [14]. In 2022, Taiwan’s National Health Insurance incurred expenditures totaling NT$8.775 billion (≈USD292 million) for rare diseases [15], placing a heavy burden on the medical system.

Current management of Von Hippel–Lindau (VHL) disease remains primarily medically oriented, with psychological support, long-term counseling, and patient-centered resources still limited. Individuals with rare diseases frequently experience prolonged diagnostic delays, fragmented care, and restricted access to reliable medical information, all of which contribute to uncertainty and psychological distress [16]. Despite these challenges, empirical research examining the psychosocial experiences and support needs of VHL patients—particularly those with central nervous system hemangioblastomas—remains extremely scarce. Approximately 40% of patients report clinically significant psychological distress, yet only one-third receive professional psychosocial support, underscoring the often-overlooked long-term burden on patients and their families [17]. Although recent advances in diagnosis and treatment have improved clinical outcomes, existing literature continues to focus predominantly on molecular mechanisms, clinical manifestations, and therapeutic results, with minimal attention to patients’ emotional responses, feelings of powerlessness, or unmet support needs. Therefore, this study aimed to explore, from the patient perspective, the lived experiences and psychological responses of individuals with VHL disease accompanied by CNS hemangioblastomas, to clarify their emotional challenges, coping strategies, and unmet care needs, and to address the psychosocial gaps in the current literature. The findings may inform the development of patient-centered support interventions and provide, an empirical foundation for policymakers to establish more integrated and patient-centered models of rare disease care.

## 2. Materials and Methods

### 2.1. Research Setting and Participants

This study was conducted in the neurosurgical outpatient clinic of a medical center in Taipei, Taiwan, that serves as a referral hospital for patients with rare diseases and provides long-term, multidisciplinary care for individuals with VHL disease. Participants were recruited using purposive sampling. The inclusion criteria were as follows: (1) being aged 18 years or older, (2) having received a diagnosis of VHL disease with CNS hemangioblastomas from a physician, and (3) being able to communicate in Mandarin or Taiwanese. Individuals diagnosed with dementia, delirium, or psychiatric disorders were excluded. Participant recruitment and data analysis were concurrently conducted. When thematic categories began to repeat with no emergence of new perspectives, data were considered saturated, and recruitment was discontinued. In total, 12 patient interviews were completed.

### 2.2. Research Instruments

The research instruments comprised an interview guide, a digital voice recorder, and reflective notes. The research team comprised four professionals: a qualitative research expert; a clinical nurse specialist with extensive experience in rare disease care; a neurosurgeon specializing in central nervous system tumors; and a nursing faculty member with expertise in psychosocial research. All interviews were conducted by the first author, a senior clinical nurse specialist with over 10 years of experience in neurosurgical and rare disease care, who has received formal training in qualitative interviewing. The researcher has 37 years of clinical nursing experience and independently conducted all face-to-face in-depth interviews. A semi-structured interview guide was initially developed by the research team based on their clinical experience and a review of existing qualitative studies on rare diseases and was subsequently revised according to the results of a pilot study involving two participants. The finalized interview guide comprised seven open-ended questions (Table 1).

### 2.3. Data Collection Procedure

This study was approved by the institutional review board of a medical center in Taipei, Taiwan (IRB no.: CGH-P112021) and was conducted in accordance with the principles of the Declaration of Helsinki. Eligible and willing participants were recruited after the researcher explained the study’s purpose, procedures, and participants’ rights; each participant then signed an informed consent form. The recruitment period spanned from July 2023 to June 2024. Interviews were conducted in a quiet consultation room within the neurosurgery department of the medical center. All interviews were conducted by a single trained qualitative interviewer, who maintained a neutral stance and used a semi-structured interview guide and probing/communication techniques to facilitate in-depth descriptions while minimizing interviewer influence. With participants’ permission, interviews were audio-recorded using a digital voice recorder. The researcher also documented field notes on participants’ facial expressions and body language and maintained reflective notes throughout the data collection process. Each interview lasted 15–60 min, depending on participants’ clinical condition, fatigue level, emotional state, and narrative flow. Interview length varied among participants, as some required shorter sessions due to physical discomfort or fatigue, while others, being in more stable condition, were able and willing to provide more detailed accounts. Audio recordings were transcribed verbatim within 72 h. Transcripts were repeatedly reviewed and verified against the recordings prior to analysis, and data analysis proceeded concurrently with transcription.

### 2.4. Data Analysis

This study employed a content analysis [17,18] to analyze the transcribed interview data. This analysis employed the following steps: (1) the researcher repeatedly read the transcripts and made detailed annotations to identify initial meaning units; (2) the meaning units were condensed and categorized for coding; (3) provisional themes or categories were defined; (4) the categories were reexamined and reorganized for repeated trial coding until all meaning units were appropriately classified; and (5) two research team members compared the coding results to ensure consistency in interpretation and establish coherent categories and themes. Themes and subthemes were allowed to emerge organically from participants’ narratives rather than being constrained by an a priori theoretical structure, thereby ensuring that the findings authentically reflected patients’ lived experiences.

The researcher also documented personal reflections and insights throughout the data collection process. To minimize potential bias and enhance research credibility, the research team maintained a reflexive journal throughout the study and engaged in regular interdisciplinary discussions to critically reflect on preliminary analytical findings.

The ongoing clarification of concerns with the participants strengthened the confirmability of the data analysis and results. To ensure dependability, all interviews were conducted by a single researcher, ensuring consistency in data collection. Verbatim transcription of each interview was completed within 72 h, and the researcher continually reviewed the transcripts to confirm the transcription accuracy.

### 2.5. Research Rigor

The rigor of the study was based on the methods of Lincoln and Guba [19]. The study was conducted by a researcher with extensive clinical experience and formal training in qualitative research. All interviews were audio-recorded and transcribed verbatim, and the transcripts were repeatedly reviewed and verified before analysis. To enhance the credibility of the findings, peer researchers and experts with qualitative research backgrounds were invited to discuss and review the results. The researcher used a semi-structured interview guide and various communication techniques to elicit in-depth, multifaceted descriptions of participants’ experiences. Detailed descriptions of the research setting, inclusion and exclusion criteria, and participant recruitment methods were provided to achieve thick description, supporting the transferability of the findings to similar contexts. During the interviews, the researcher maintained a neutral and objective stance to avoid influencing the participants’ expression of their views. Subjective assumptions were minimized, and participants were encouraged to freely share their thoughts.

## 3. Results

A total of 12 participants were recruited (8 women, 4 men). The mean age was 52.3 years (SD = 15.3). Most participants had completed high school (50%) or college (33.3%), were married (83.3%), and identified as Buddhist (83.3%). The median duration of illness was 23 years (IQR = 4–32), and the median number of major surgeries was 1 (IQR = 1–2). The majority of participants were fully independent in functional status (91.7%), with only one participant being partially dependent (Table 2). Using a content analysis, the researcher categorized illness experiences of participants with VHL disease and CNS hemangioblastomas into four main themes: powerlessness, negative emotional experiences, compromise, and persistent worry. Each theme consisted of two to three subthemes. Table 3 contains the themes, subthemes, and example quotes from interviewees.

### 3.1. Theme 1: Powerlessness

Faced with the unpredictable and variable progression of VHL disease, participants were experiencing a profound sense of powerlessness. Unlike conditions that can be managed or stabilized through medical interventions, VHL disease is a hereditary disorder characterized by an uncontrollable and indefinite course. The uncertainty surrounding when or if the disease would become terminal led participants to feel a loss of control over their bodies and daily life, causing them to experience a strong sense of powerlessness.

***Unpredictable disease progression***

VHL disease is rare and presents with diverse manifestations. Patients must endure prolonged periods of unexplained physical abnormalities without receiving a definitive diagnosis. The unpredictable and variable nature of the disease trajectory caused the participants ongoing physical and psychological distress.

***Uncontrollable continuity***

In contrast to common illnesses that can be managed through medical treatment, VHL disease is hereditary and characterized by an unpredictable and unrelenting progression. Patients are unable to foresee the course or outcome of their illness, and often feel trapped in a prolonged and uncontrollable journey.

### 3.2. Theme 2: Negative Emotional Experiences

Due to the unpredictable and variable nature of VHL disease, participants expressed a range of negative emotions. These included feelings of guilt toward their families and self-blame, as well as depression. Some participants also exhibited diminished self-worth. These emotions were conveyed verbally and through nonverbal behaviors such as sobbing, frowning, and crying.

***Guilt and self-blame***

Several participants expressed strong feelings of guilt and self-blame when narrating the course of their illness and its hereditary nature. They felt remorseful not only for the burden their illness imposed on their families but also for the possibility of having passed the condition on to the next generation.

***Depression***

When recounting their disease trajectories, the participants frequently revealed feelings of depression, pessimism, and suffering. Some expressed grief and a sense of loss due to family members also being affected by VHL disease. Others described feelings of helplessness and despair in response to recurrent symptoms or limited treatment options.

***Low self-esteem***

The progression of VHL disease affected the participants physically and substantially influenced their self-evaluation and emotional well-being. Confronted with misunderstanding or dismissal from others as well as physical limitations imposed by the disease, several participants exhibited a diminished sense of self-worth.

### 3.3. Theme 3: Compromise

In the face of uncertainty and the unpredictable nature of VHL disease, several participants learned to compromise. They gradually relinquished the desire to control their fate and began striving to coexist with their illness. The participants not only accepted the physical changes and challenges associated with treatment but also learned to accept the course of their condition. As they sought support from medical and social resources, they learned to face adversity with resilience and adapted their lifestyles accordingly. Simultaneously, they expressed hope that advances in medical technology would lead to improved disease management and that a sound social support system could be established.

***Acceptance of one’s fate***

Over the course of living with VHL disease, several participants gradually relinquished the expectation of complete recovery and learned to accept the changes occasioned by their illness. They understood that certain aspects of their condition were beyond their control and that only by adjusting their mindset and accepting their fate could they reduce their suffering and regain a sense of balance in life.

***Positive outlook***

Focuses on how one faces the illness in the present. Despite the numerous challenges posed by VHL disease, some participants learned to face their condition with a positive mindset. They believed that rather than dwelling on negative emotions, they should constructively seek ways to improve their quality of life and engage proactively in treatment and rehabilitation. This kind of positive thinking helped them stay motivated and better adapt to changes in their condition.

***Sense of hope***

Focuses on future possibilities. It highlights expectations and confidence regarding future health, medical advances, and social support, maintaining the strength to look forward even in the face of uncertainty. Amid the prolonged suffering and uncertain future occasioned by VHL disease, participants continued to exhibit hope and confidence in the future. Although their hope was often tempered by the realities of hardships and unpredictability, they did not relinquish their desire for improved health. They also expressed hope for improvements and support from both the medical system and broader society. Their hope was anchored in the advancement of medical technology and the development of robust social support systems that would enable individuals living with rare diseases to receive more substantial assistance.

### 3.4. Theme 4: Persistent Worry

For patients with VHL disease accompanied by CNS hemangioblastomas, the illness imposes not only physical burdens but also extends a psychological burden throughout the family system because of its hereditary nature. The condition may be inherited from the previous generation and transmitted to the next, and this potential intergenerational continuity generates persistent worry for relatives and children, creating an ongoing psychological weight that is difficult to remove. The unceasing course of the disease and its treatment also affects personal career prospects and financial stability, heightens anxiety about life’s unpredictability, and leaves patients filled with uncertainty about the future.

***Worries about family members***

The hereditary nature of VHL disease leads patients to bear both the burden of their own illness and deep concerns for their family members. Some of the participants expressed intense anxiety over the possibility of transmitting the condition to the next generation. For those who were parents, this concern often evolved into an ongoing struggle with decisions regarding life planning and family arrangements.

***Anxiety regarding finances and employment***

Living with VHL disease and CNS hemangioblastomas requires patients to undergo long-term treatment and multiple surgeries. This treatment places a considerable physical burden on patients and affects their family’s financial structure by limiting patients’ ability to work. For most participants, the disease marked a turning point that necessitated a redefinition of their breadwinning role within the household. This adjustment often occasioned intense anxiety regarding reduced income and increased financial hardships.

***Uncertainty regarding the future***

VHL disease imposes long-term and unpredictable effects on patients’ career development and daily lives. Participants consistently reported that neurological impairments, limitations in physical mobility, and persistent physical discomfort hindered their ability to engage in work and social activities. As a result, they experienced a growing sense of uncertainty and confusion regarding their future.

## 4. Discussion

In this qualitative study, we conducted an in-depth analysis of the illness experiences of patients with VHL disease accompanied by CNS hemangioblastomas. Findings were categorized across four major themes: powerlessness, negative emotional experiences, compromise, and persistent worry. Peng et al. [20] pointed out that patients with rare diseases often experience helplessness and emotional distress during their prolonged and uncertain diagnostic journeys. Delays in diagnosis and insufficient information can lead to heightened anxiety and self-doubt, reflecting common problems of knowledge gaps and inadequate support regarding rare diseases within the healthcare system. These observations resonate with the accounts of the participants in the present study, who described feelings of anxiety, powerlessness, and uncertainty regarding the future as they awaited a diagnosis and monitored recurrence.

Powerlessness refers to the frustration and despair experienced by patients when faced with an illness whose outcomes they cannot alter or control. VHL disease is a rare autosomal dominant hereditary condition with a 50% inheritance rate that requires patients to undergo lifelong monitoring for tumors that may develop across multiple organ systems [21,22]. In this study, several participants described being unable to predict when their tumors would recur, likening the experience to a sudden and silent assault on their body. The disease’s manifestations—such as neurological damage and sensory abnormalities (e.g., sensations of ants crawling on the body or of floating)—emerged unpredictably, and before the participants had received a definitive diagnosis, the unpredictable nature of symptoms (including headaches, ataxia, loss of balance, and pain or numbness in the back, arms, and legs) rendered the participants’ bodies unfamiliar and uncontrollable. Moreover, participants observed that even after they had received a diagnosis, they could only attempt to manage the disease because they had no prospect of a cure. Combined with the inability to prevent transmission of the condition to the next generation, this lack of hope for a cure fostered an overwhelming sense of powerlessness.

The second theme identified in this study was participants’ negative emotional experiences. Participants expressed feelings such as guilt, depression, and low self-esteem, echoing the findings of research on patients with VHL disease, indicating that insufficient medical resources and a lack of social support frequently lead patients to experience emotional breakdowns [23]. In their narratives of guilt and self-blame, participants described feeling trapped between death and paralysis, worried about becoming burdens to their families, regretful of failing to fulfill expected family roles, and concerned regarding the possibility of transmitting the disease to their children. Participants’ frequent crying and nonverbal behaviors—such as clenching or squeezing their hands—highlighted their uncontrollable feelings of depression and vividly demonstrated the intensity of their emotions. Rezaei et al. [22] reported that some patients with rare diseases expressed disappointment with the healthcare system because of their frequent medical visits and unresolved symptoms. Additionally, changes in physical appearance and function lead to reduced self-esteem and social isolation, paralleling the experiences described by participants in this study, who faced social stigma, feelings of inferiority regarding their appearance, and a reluctance to leave their home. In addition, Pelentsov et al. [24] similarly noted that insufficient support during prolonged caregiving contributed to feelings of isolation, breakdown, and loss, negatively affecting the psychological health of both patients and their families. Currie and Szabo [25] also reported that inadequate medical knowledge, scarce social resources, and heavy caregiving responsibilities resulted in profound feelings of isolation and alienation. Although their study primarily focused on family caregivers, experiences of social exclusion and isolation they identified closely resembled the negative emotional responses of participants in the present study.

Compromising and coexisting with the disease emerged as central coping strategies among participants. Rather than a passive concession, compromise functioned as a dynamic adaptive process through which participants transformed feelings of powerlessness and loss of control into acceptance, identity redefinition, and cautious hope—reflecting both resilience and post-traumatic growth. This process aligns with Southwick et al.’s interdisciplinary resilience framework [26], which emphasizes meaning-making, agency, and psychological flexibility in the context of chronic adversity. Resilience among patients with rare diseases is not static but gradually shaped through long-term interaction with illness. Consistent with the previous study, participants demonstrated resilience by reassessing the meaning of their disease and integrating it into their life narratives [27]. In the interviews, they acknowledged the disease’s impact while emphasizing that a hopeful and meaningful life remained possible. Despite inevitable discouragement and emotional distress, participants chose to seek treatment and actively confront their condition. Some interpreted the illness as a test, reflecting a rational and pragmatic stance, and reframed it as a surmountable challenge rather than a terminal condition. After enduring critical episodes, several participants expressed gratitude for life and developed a more optimistic outlook. Although they recognized the heavy physical and psychological burden of VHL and the low likelihood of a cure, they maintained rational hope—trusting advances in medical technology to help control disease progression. Many also expressed a strong desire not to pass the disease on to the next generation and remained hopeful about the support of family, society, and future medical developments. This cautious optimism illustrates a balanced form of hope, neither naïve nor resigned. These findings echo Zhou et al.’s [28] assertion that strengthening hope enhances resilience in patients and caregivers, as well as Frank’s [29] observation that individuals with chronic or rare diseases reconstruct self-identity and life meaning through everyday practices and narrative expression. Over time, participants moved from denial and resistance toward acceptance and coexistence with VHL, marking a difficult yet authentic psychological transformation grounded in resilience and meaning-making.

For participants in this study, persistent worry was a lifelong challenge. Due to the highly hereditary nature of von Hippel–Lindau (VHL) disease, patients not only endured their own physical suffering but also expressed concerns for the health of their children, spouses, and elderly parents, highlighting the impact of the disease on family roles, emotional responsibilities, and intergenerational relationships. In addition, participants faced financial pressures from medical expenses and reduced earning capacity, which were further exacerbated by insufficient social support, uncertainty regarding life after diagnosis, and progressive physical deterioration. These findings are consistent with previous research indicating that rare diseases negatively affect patients’ quality of life, social functioning, and ability to manage multiple simultaneous challenges [30,31]. Family systems theory (FST) posits that the family is an interdependent system in which changes in one member influence the functioning, roles, and well-being of other members [32]. In the context of rare diseases, intergenerational worries—parents’ or caregivers’ concerns regarding disease inheritance, children’s future health, and family continuity—are systemic phenomena embedded within family interactions rather than isolated psychological responses. Such worries influence caregiving strategies, emotional expression, and risk communication, shaping children’s understanding of their illness and long-term expectations, and may also be transmitted across generations through family narratives [33,34]. Applying FST provides insight into how intergenerational worries can be amplified or mitigated within family systems and supports interventions that shift from an individual-centered to a family- and intergenerational-centered approach [35,36].

In addition, we also found that disease duration influenced emotional responses. Participants with longer disease durations typically described an emotional trajectory that evolved from initial fear, confusion, and loss of control to more adaptive attitudes, such as acceptance, compromise, and cautious hope. In contrast, those who were more recently diagnosed or had shorter disease durations more often expressed anxiety, uncertainty, and a sense of loss. With regard to gender, female participants more frequently expressed concerns related to caregiving responsibilities and family continuity, whereas male participants tended to emphasize financial pressure and employment-related worries. Given the small sample size, these observations are exploratory, but they suggest that gendered expectations may subtly influence patients’ interpretation and narrative of their disease experiences. Therefore, future research is recommended to further explore gender differences in the adaptation process of rare genetic diseases.

Furthermore, we also discovered religious and spiritual beliefs, especially Buddhism, stood out as important coping resources in participants’ narratives and played a significant role in shaping acceptance, emotional regulation, and meaning construction within the context of VHL. Some participants used Buddhist concepts of impermanence, karma, and letting go to reinterpret the unpredictability of the disease, which helped them transform self-blame and despair into a more accepting and balanced attitude, demonstrating that religion is an important psychological resource in chronic disease. A recent systematic review showed that higher spiritual well-being and religious coping were closely associated with improved quality of life, reduced psychological distress, and enhanced coping abilities in cancer patients [37]. Spirituality is not only a belief system but also a dynamic process through which patients reinterpret adversity and maintain psychological resilience.

## 5. Conclusions

This study identified four major themes in the illness experiences of patients with VHL disease accompanied by CNS hemangioblastomas: powerlessness, negative emotional experiences, compromise, and persistent worry. Patients with VHL disease face the risk of permanent disability due to organ dysfunction and physical impairments caused by the illness, which adversely affect their personal lives and employment and pose challenges to family dynamics and overall social productivity. By analyzing the perspectives of participants, this study offers a detailed depiction of cognitive responses, emotional trajectories, and coping strategies in the face of illness, providing healthcare professionals with deep insights into the care needs of individuals with VHL disease. In addition to delivering standard medical services, healthcare providers should serve as sources of emotional support and companionship—actively listening to patients’ needs and providing reliable mental health resources and information regarding VHL disease to reduce the anxiety and distress caused by a lack of knowledge.

Furthermore, based on the findings of this study, we propose the following recommendations for future practice and policy. (1) Establish multidisciplinary integrated care models by forming patient-centered teams that include neuro-oncologists, genetic counselors, relevant subspecialists, nurses, mental health professionals, social workers, and case managers. Such teams can comprehensively support patients and their families in genetic screening, early detection and treatment, and in coping with disease-related uncertainty, genetic anxiety, and life-adjustment challenges. (2) Strengthen family-oriented care by incorporating systematic genetic surveillance and standardized psychosocial assessments into routine follow-up for patients with VHL disease. (3) Develop peer-support and networking platforms through collaboration between healthcare institutions and rare disease foundations. Establishing in-person or online peer-support groups can facilitate experience sharing, reduce social isolation, and enhance patients’ coping capacities. (4) Enhance integration between social welfare resources and treatment policies. We recommend that relevant authorities expedite the evaluation of accessibility and reimbursement feasibility for novel therapies—such as the oral HIF-2α inhibitor belzutifan—in Taiwan and coordinate these efforts with social welfare resources to provide more comprehensive and sustainable support for patients.

## 6. Strengths and Limitations of the Study

This study is the first in-depth qualitative investigation to date to explore the illness experiences of patients with VHL disease accompanied by CNS hemangioblastomas. Although the number of interviews was limited, more than half of the participants had been living with the disease for more than 20 years and had developed long-standing, trusting relationships with healthcare providers. These factors contributed to the richness and authenticity of their narratives. The rich data and findings of this study offer valuable insights for the clinical care and support of patients affected by this rare condition.

Because of the extreme rarity of VHL disease, this study included only 12 participants, a low number that represents this study’s most substantial limitation. Potential selection bias from recruiting from a single center and the notable absence of family caregiver perspectives, which limits the understanding of the familial care dynamic. Future studies should recruit larger and more diverse samples across multiple healthcare institutions to improve generalizability. Incorporating family members’ perspectives may further strengthen family support and interdisciplinary collaboration, supporting the development of a family-centered holistic care model for individuals with VHL and their families.

## Figures and Tables

**Table 1:** Interview guide.

1. How were you diagnosed with VHL disease and central nervous system hemangioblastomas? What are your thoughts about having this condition?
2. How has this disease affected your life?
3. How does having VHL disease with central nervous system hemangioblastomas make you feel?
4. What worries you about this disease?
5. During the process of managing your illness, have you encountered any difficulties? Are there any ways you think these challenges could be improved?
6. Overall, what does having this disease mean to you?
7. In addition to what you have already shared, is there anything else you would like to add?

**Table 2:** Socio-demographic and clinical characteristics of participants (N = 12).

Characteristic	Summary	
Sex, n (%)	Male	4 (33.3%)
	Female	8 (66.7%)
Age (years)		Mean ± SD: 52.3 ± 15.3 Range: 29–71
Educational level, n (%)	Elementary school	1 (8.3%)
	Junior high school	1 (8.3%)
	High school	6 (50.0%)
	College	4 (33.3%)
Marital status, n (%)	Married	10 (83.3%)
	Unmarried	2 (16.7%)
Religion, n (%)	Buddhist	10 (83.3%)
	None	2 (16.7%)
Duration of illness (years)		Median (IQR): 23 (4–32) Range: 1–34
Number of major surgeries		Median (IQR): 1 (1–2) Range: 1–6
Functional status, n (%)	Fully independent	11 (91.7%)
	Partially dependent	1 (8.3%)

**Table 3:** Theme and Subthemes with Exemplary Quotes from the Interviewees.

Theme	Subtheme	Exemplary Quotes
Powerlessness	Unpredictable disease progression	*At first, it was a numb sensation on my thumb, and then it spread to my ear. The numb area was about the size of a NT$10 coin, and it felt funny to the touch. I couldn’t hold things with my left hand at all. I ended up breaking all the plates in my house. (Case 6)* *My hands felt strange. Even though I had washed them thoroughly, they still felt greasy. It was as if my hands were plates used for oily stir-fried vegetables. No matter how hard I tried to clean them, they still felt greasy to the touch. This unusual foreign-body sensation was present everywhere. I also felt as though ants were crawling on me. My feet felt odd when I walked, as if there were foreign objects embedded in them. My feet also experienced a sense of swelling, like wearing air-cushioned shoes. At times, my tendons felt very tight, as if I could not exert strength, and my lower back also ached. (case 8)*
	Uncontrollable continuity	*I do not want my family to end up like me because of this. I hope it will not be passed on to the next generation. I truly hope there will be effective medication to treat this disease so that it does not persist. (case 2)* *I’m worried that it might recur. I’ve heard that hemangioblastomas can grow back, like grapes—they just keep growing more and more, never less (frowning). (case 11)*
Negative emotional experiences	Guilt and self-blame	*I feel so sad… I have not been able to work since I was young—my husband has been the one taking care of me… (crying…) And now my child has it too (inherited the condition). My mother also feels very sad and blames herself because the gene that affects us comes from her side of the family (crying…). (case 2)* *Actually, I am not afraid of dying; I am afraid of not dying. If I become paralyzed… I … I would end up burdening my family because of my illness (crying…). (case 4)*
	Depression	*My older brothers—I think they had this disease too. They underwent surgery on the back of their heads. It was painful and they became very depressed. Eventually, they passed away (eyes reddening…). (case 3)* *When it was at its worst, I was crying all the time. The more I cried, the louder I got, and the more heartbroken I felt. I would cry for no apparent reason. (case 6)*
	Low self-esteem	*Some of my relatives really look down on me. They say I am faking it—probably because you can’t see it from the outside, since I can still walk and cook. (case 6)* *My thoughts about this disease are just—how unlucky I am. My limbs are weak, my body does not respond the way I want it to, I need a cane to support myself, and I walk very slowly. It’s like I’ve had a stroke. I don’t really dare go outside anymore. I just stay at home all the time. (case 2)*
Compromise	Acceptance of one’s fate	*To live with it—that’s what I chose. Over time, I slowly adjusted my mindset and came to accept it. I sought comfort through religious faith and increased interactions with others, and that helped me gradually reshape my attitude. (case 12)* *I started doing rehabilitation little by little—because in the end, I could only rely on myself. What else could I do? This disease just came out of nowhere. (case 5)*
	Positive outlook	*I am pretty brave when it comes to facing my illness. My husband said if it could be treated or operated on, then I should go seek medical attention right away. I believe everyone gets sick at some point. Since it happened to me, as long as surgery can help me recover and not affect my life, I will face it with courage. (case 2)* *I consider myself a healthy person. This illness will not defeat me. I don’t even see it as a disease. I believe that if a tumor appears, it has to be surgically removed so I can recover. So, I face it with courage. I don’t dwell on anything. Of course, after the brain surgery, I did think about the possible bad outcomes, since it was my first brain operation and a huge challenge. But I told myself: it happened, so face it. (case 4)*
	Sense of hope	*I hope I can become healthier. I do not want my family to be affected by this—I don’t want it to be passed on to the next generation. I hope that one day there will be effective medication to treat this disease. (case 4)* *I hope that in the future, the National Health Insurance system or social resources can provide better medical care and support for people with rare diseases. Some medications are not covered by insurance and must be paid out of pocket. I tried to apply for subsidies, but the requirements are very strict. For example, I could not even get assistance for my visual impairment—the eligibility threshold was just too high. (case 9)*
Persistent worry	Worries about family members	*Actually, I am not afraid of dying; I am afraid of not dying. If I become paralyzed… I … because I… (crying…) I have three children… (crying…) and they are still young. (case 2)* *After getting married, I started to question whether I should become pregnant or have children. This is a genetic disease, and I had already undergone three surgeries before having children. So, I was worried—worried about my own health and about whether it would affect my children. If this problem does not have a good resolution, I don’t think I will dare to have children again. (case 8)*
	Anxiety regarding finances and employment	*If the surgery does not go well, it could affect my ability to work, and that would tighten our finances. I am hoping to get some government assistance, because after surgery, I might need several months to recover. My company cannot accommodate such a long leave, so I am really hoping for financial support—after all, I have a family to support. (case 1)* *My concern about work is really about how it affects the family’s finances. We have two children to raise, and money has to be used wisely. Back when both my spouse and I were working, we felt we had everything we needed. But ever since I stopped working, financial pressure has been present. (case 6)*
	Uncertainty regarding the future	*Will I have to live with these tumors for the rest of my life? I was diagnosed at 30, and now I am 60—I have coexisted with them for 30 years. But now I am getting old, and so are my body and nervous system. The pain from this disease—will it just keep getting stronger and stronger? It weighs on me. The numbness in my hand makes everyday life difficult. This disease is with me 24 h a day without pause. (case 2)* *After surgery, I could no longer take on large-scale construction projects like I used to because of a lack of stamina. I shifted to doing small-scale projects. My thoughts were quite jumbled back then. After being diagnosed with this disease, I didn’t know what to do. And so one year passed, then another… and many more after that. (case 10)*

## Data Availability

The raw data supporting the conclusions of this article will be made available by the authors on request.

## Abbreviations

The following abbreviations are used in this manuscript:

VHL	Von Hippel–Lindau
CNS	Central nervous system

## References

[1] Jonasch E., Song Y., Freimark J., Berman R., Nguyen H., Signorovitch J., Sundaram M. (2023). Epidemiology and Economic Burden of von Hippel-Lindau Disease-Associated Renal Cell Carcinoma in the United States. Clin. Genitourin. Cancer.

[2] Gossage L., Eisen T., Maher E.R. (2015). VHL, the story of a tumour suppressor gene. Nat. Rev. Cancer.

[3] Yin X., Duan H., Yi Z., Li C., Lu R., Li L. (2020). Incidence, prognostic factors and survival for Hemangioblastoma of the central nervous system: Analysis based on the surveillance, epidemiology, and end results database. Front. Oncol..

[4] Páleníková P., Adamcová M., Šturdík I., Ftáčniková B., Copáková L., Payer J. (2016). Von Hippel-Lindau syndrome-two sides of the same coin. Vnitr. Lek..

[5] Ikeuchi Y., Nishihara M., Ashida N., Hosoda K. (2022). Von Hippel-Lindau disease with intracranial hemorrhage due to arteriovenous anastomosis via multiple spinal hemangioblastomas: Illustrative case. J. Neurosurg. Case Lessons.

[6] Witt S., Schuett K., Wiegand-Grefe S., Boettcher J., Quitmann J. (2023). Living with a rare disease-experiences and needs in pediatric patients and their parents. Orphanet J. Rare Dis..

[7] Kreatsoulas D.C., Lonser R.R. (2024). Spinal cord hemangioblastomas in von Hippel–Lindaudisease. Neuro-Oncol. Adv..

[8] Huntoon K., Shepard M.J., Lukas R.V., McCutcheon I.E., Daniels A.B., Asthagiri A.R. (2022). Hemangioblastoma diagnosis and surveillance in von Hippel-Lindau disease: A consensus statement. J. Neurosurg..

[9] Louise M., Binderup M., Smerdel M., Borgwadt L., Nielsen S.S.B., Madsen M.G., Møller H.U., Kiilgaard J.F., Friis-Hansen L., Harbud V. (2022). von Hippel-Lindau disease: Updated guideline for diagnosis. Eur. J. Med. Genet..

[10] Launbjerg K., Bache I., Galanakis M., Bisgaard M.L., Binderup M.L.M. (2017). von Hippel-Lindau development in children and adolescents. Am. J. Med. Genet..

[11] Lammens C.R., Bleiker E.M., Verhoef S., Hes F.J., Ausems M., Majoor-Krakauer D., Sijmons R.H., Van Der Luijt R.B., Van Den Ouweland A.M., Van Os T. (2010). Psychosocial impact of Von Hippel–Lindau disease: Levels and sources of distress. Clin. Genet..

[12] Hwu W.L., Hsu R.H., Li M.H., Lee H.M., Chen H.A., Lee N.C., Chien Y.-H. (2023). Aromatic l-amino acid decarboxylase deficiency in Taiwan. JIMD Rep..

[13] Health Promotion Administration, Ministry of Health and Welfare (2024). Prevention and Treatment of Rare Diseases.

[14] Health Promotion Administration, Ministry of Health and Welfare (2025). Statistical Report of Rare Disease Confirmed Cases in Taiwan (December 2024).

[15] Ministry of Health and Welfare (2025). The National Health Insurance Administration Is Fully Committed to Caring for Patients with Rare Diseases, Leaving No One Behind and Making Every Effort to Negotiate with Pharmaceutical Companies. https://www.mohw.gov.tw/cp-16-73228-1.html.

[16] Anderson M., Elliott E.J., Zurynski Y.A. (2013). Australian families living with rare disease: Experiences of diagnosis, health services use and needs for psychosocial support. Orphanet J. Rare Dis..

[17] Graneheim U.H., Lundman B. (2004). Qualitative content analysis in nursing research: Concepts, procedures and measures to achieve trustworthiness. Nurse Educ. Today.

[18] Liang S.Y., Chuang Y.H., Wu S.F. (2012). Preliminarily Application of Content Analysis to Qualitative Nursing Data. J. Nurs..

[19] Lincoln Y.S., Guba E.G. (1985). Establishing trustworthiness. Nat. Inq..

[20] Peng C.Y., Liu M.P., Lee P.H., Yang C.I., Hsieh H.T., Hsieh M.C., Lee L.H. (2023). The Health-Seeking Experience of Patients with Sjögren’s Syndrome. J. Nurs..

[21] Chang M.L., Sun L.C., Tsai P.H. (2017). Nursing Experience of Caring for a Middle-Aged Woman with Brain Tumor. Kaohsiung J. Nurs..

[22] Rezaei F., Sanagoo A., Peyrovi H., Jouybari L. (2023). Persistent suffering: Living experiences of patients with rare disease: An interpretative phenomenological study. J. Educ. Health Promot..

[23] Wolters W.P.G., Dreijerink K.M.A., Giles R.H., van der Horst-Schrivers A.N.A., van Nesselrooij B., Zandee W.T., Timmers H.J., Seute T., de Herder W.W., Verrijn Stuart A.A. (2022). Multidisciplinary integrated care pathway for von Hippel–Lindau disease. Cancer.

[24] Pelentsov L.J., Fielder A.L., Esterman A.J. (2016). The Supportive Care Needs of Parents with a Child with a Rare Disease: A Qualitative Descriptive Study. J. Pediatr. Nurs..

[25] Currie G., Szabo J. (2020). Social isolation and exclusion: The parents’ experience of caring for children with rare neurodevelopmental disorders. Int. J. Qual. Stud. Health Well-Being.

[26] Southwick S.M., Bonanno G.A., Masten A.S., Panter-Brick C., Yehuda R. (2014). Resilience definitions, theory, and challenges: Interdisciplinary perspectives. Eur. J. Psychotraumatol..

[27] Schwartz C.E., Michael W., Rapkin B.D. (2022). Correction to: Resilience to health challenges is related to different ways of thinking: Mediators of physical and emotional quality of life in a heterogeneous rare-disease cohort. Qual. Life Res..

[28] Zhou M., Wang M., Luo D., Sun C., Bian Q., Xu J., Lin Z. (2024). The mediating role of resilience between caregiver burden and hope among patients with inflammatory bowel disease. Nurs. Open.

[29] Frank A.W. (1995). The Wounded Storyteller: Body, Illness, and Ethics.

[30] Chateau A.V., Blackbeard D., Aldous C. (2023). The impact of epidermolysis bullosa on the family and healthcare practitioners: A scoping review. Int. J. Dermatol..

[31] von der Lippe C., Neteland I., Feragen K.B. (2022). Children with a rare congenital genetic disorder: A systematic review of parent experiences. Orphanet J. Rare Dis..

[32] Bowen M. (1993). Family Therapy in Clinical Practice.

[33] McCarthy S.R., Golembiewski E.H., Gravholt D.L., Clark J.E., Clark J., Fischer C., Mulholland H., Babcock K., Montori V.M., Jones A. (2022). Documentation of Psychosocial Distress and Its Antecedents in Children with Rare or Life-Limiting Chronic Conditions. Children.

[34] Domaradzki J., Walkowiak D. (2024). Invisible patients in rare diseases: Parental experiences with the healthcare and social services for children with rare diseases. A mixed method study. Sci. Rep..

[35] Smith S.R., Hamon R.R. (2022). Exploring Family Theories.

[36] Joseph B., Dickenson S., McCall A., Roga E. (2023). Exploring the therapeutic effectiveness of genograms in family therapy: A literature review. Fam. J..

[37] Nagy D.S., Isaic A., Motofelea A.C., Popovici D.I., Diaconescu R.G., Negru S.M. (2024). The Role of Spirituality and Religion in Improving Quality of Life and Coping Mechanisms in Cancer Patients. Healthcare.

